# It Is Not Simply Black and White: A Qualitative Study Unpacking How Women Understand Racism and Discrimination

**DOI:** 10.1007/s40615-025-02552-0

**Published:** 2025-07-21

**Authors:** Shaniece Criss, Nhung Thai, Monica M. De La Cruz, Katrina Makres, Melanie Kim, Thu T. Nguyen

**Affiliations:** 1 Department of Health Sciences, Furman University, Greenville, SC 29613 USA; 2 Department of Nutritional Sciences and Toxicology, University of California, Berkeley, CA 94720 USA; 3 School of Social Welfare, University of California, Berkeley, CA 94720 USA; 4 Department of Epidemiology & Biostatistics, School of Public Health, University of Maryland, College Park, MD 20742 USA; 5 Department of Anthropology, Brown University, Providence, RI 02912 USA

**Keywords:** Black women, White women, Racism, Allyship, Qualitative research

## Abstract

The aim of this exploratory focus group study is to examine how Black women, White women, and White women involved in an anti-racism group perceive race and racism in daily life and the strategies they use or recommend to protect themselves and others. While the primary emphasis is on racial identity and racism, the analysis also considers how these dynamics intersect with gender for women across racial groups. A purposive sample of 30 participants participated in six online racially/ethnically homogeneous focus groups. A team consensus approach was utilized with codebook development and thematic analysis. The research team identified three themes: (1) the dynamic between responsibility and privilege, (2) differing experiences of womanhood, and (3) self-protection from racism and allyship from racism. Black women expressed protecting themselves and family, their intersectional identity, and the challenge of sexualization. White women discussed educating themselves about racism and privilege and the saliency of misogyny and patriarchy. White women from the anti-racism group also reported the need to support marginalized groups with presence and funding. Further research could provide insight about the type of allyship Black women desire and how White people educate themselves about these issues.

## Introduction

Racism, specifically anti-Black racism, has been a foundational element of American history, shaping a racial hierarchy that continues to influence societal structures today [1, 2]. Racism operates in multiple forms and is embedded in society through structural racism, which refers to the laws, policies, institutional practices, and societal norms that create and perpetuate systemic racial inequities and oppression [1, 3]. One example of structural racism is the systematic barriers to homeownership, such as the federal government’s practice of “redlining” predominantly non-White neighborhoods, which labeled them as high-risk and unworthy of investment. This practice not only excluded communities of color from access to mortgage loans and wealth-building opportunities but also laid the groundwork for enduring racial disparities in homeownership, credit access, and neighborhood resources [3, 4]. Another example is residential and social segregation, which continues to shape educational access, social networks, and perceptions of safety and belonging [5]. Segregation not only disadvantages communities of color but also deprives White individuals of opportunities to engage across racial lines, fostering misunderstanding, discomfort, and even fear in cross-racial interaction [6].

Interpersonal experiences of racism can be particularly damaging based on their individual expressions of racial prejudice, discrimination, or bias that occur between people, often manifesting in overt or subtle behaviors, attitudes, and interactions that reflect racial inequality [7]. Within the Black community, coping with racism can vary between addressing the offense of the act versus excusing the behavior. Coping mechanisms include racial distancing or creating distance between themselves and the other party, and racial isolation such as avoiding specific events and groups of people to protect themselves from potential future racist acts [8].

While racism has targeted and profoundly impacted Black communities, its influence extends to people of all racial backgrounds, including White individuals [9]. Policies and practices based on racial exclusion do not just harm Black people but end up hurting everyone [9]. Scholar Heather McGhee offers a powerful example in *The Sum of Us*, describing how, in the 1960 s, many public swimming pools across the USA were drained or closed altogether to prevent Black communities from sharing facilities with White people—ultimately depriving everyone of access [9]. This example underscores how the costs of racism reverberate across society, even among those who appear to benefit from it. In response to these tensions, some White individuals engage in psychological strategies to shield themselves from the discomfort of acknowledging racial inequity. Prior research has documented various mechanisms through which some White individuals deny the existence or impact of racism. These denial mechanisms include apathy rooted in color blindness, fear stemming from a fragile self-concept that feels threatened by acknowledging racism, and silence as a means of dissociating from racial stimuli [10–12].

While some White people may ignore or even perpetuate racism, there are also White people who are actively working toward anti-racism. In one study of White parent–child pairs, most parents reported communicating a color-conscious ideology to their child, such as emphasizing equal rights and acknowledging racial disparities and privilege [13]. Recognizing White racist behaviors and how racism exists at every structure of society are prominent messages of social justice organizations within the White community working toward an anti-racist society [14]. There may be a continuum of self-criticality, self-awareness, and empathy that can promote self-knowledge and self-engagement toward a White antiracist identity [15]. One process is through experiencing feelings of marginalization and moral outrage of frustration and anger leading to the development of empathy with the pain and suffering of people who are marginalized [10].

Within the discussion about race, it is valuable to bring in the perspective of gender. People who identify as women often have shared experiences including gender-based mistreatment, perceived advantage, friendship and community, caretaking, and navigating decisions related to employment and family roles [16]. In addition, Black women also report an additional experience of inner strength [16]. Related to the idea of Black women’s inner strength, one of the several complex responses to racism that Black women experience is the pressure of adhering to the Strong Black Woman (SBW) schema. The SBW schema is a reaction to societal stereotypes placed on Black women in the USA [17–22]. The SBW schema pressures Black women to suppress their emotions to not be seen as argumentative [19]. They are expected to be independent but not engage in self-care while simultaneously taking care of everyone in the community [19, 20]. An example of the SBW schema is a Black woman juggling work, caregiving, and community responsibilities while suppressing her emotions and avoiding asking for help to maintain the appearance of strength and independence. While being strong can be a positive trait, the SBW schema often places significant strain on Black women in the face of racism, promoting behavior that endures mistreatment rather than calls it out [17–22]. While the SBW schema can sometimes serve as a defense, it has deleterious consequences, especially for mental and physical health [17–22].

Gender-based discrimination is a problem that is experienced by both White and Black women. Both groups have movements to push back against oppression—feminism which has historically focused on fighting gender discrimination against White women [23], and womanism which centers experiences of discrimination against Black women, including addressing systems that impact gender, sexuality, race, nation, and class [24]. Intersectionality provides a useful framework to interpret how multiple forms of oppression (e.g., gender-based discrimination and racism) interact to create unique experiences at the intersections [25, 26] while also acknowledging that some experiences at the intersection can provide privilege [27]. These different approaches are rooted in differing experiences.

Although racism has been widely studied, few studies have examined the experiences and understandings of both Black and White women within the same focus group analysis. Most existing studies focus on a single racial group, overlooking the relational dynamics between individuals who are positioned within systems of racial privilege and those who are marginalized by them. The aim of this exploratory focus group study is to examine how Black women, White women, and White women involved in an anti-racism group perceive race and racism in daily life and the strategies they use or recommend to protect themselves and others. While the primary emphasis is on racial identity and racism, the analysis also considers how these dynamics intersect with gender for women across racial groups.

## Methods

This study is part of a larger companion study that explored the impact of online and offline racism on maternal health—including pregnancy, birthing, and child-rearing experiences—among Asian and Pacific Islander, Black, Latina, and Middle Eastern women in the USA [28]. As part of that broader study, focus groups were also conducted with White women using the same interview guide. While the maternal health–related findings from communities of color were published in the companion paper, the current paper analyzes a distinct subset of data focused specifically on race and racism more broadly, rather than exclusively within maternal health contexts. To that end, a series of sub-questions addressing broader experiences with race and racism were embedded in the original interview guide and asked of all focus groups, regardless of racial/ethnic identity.

The present analysis draws on six focus groups (three with Black women and three with White women, including two composed of White women engaged in anti-racism work), with a total of 30 participants. Participants were eligible if they (1) identified as a woman (inclusive of cisgender and transgender identities), (2) were at least 18 years old, (3) identified as Black or White, (4) used social media, (5) had children or were open to having children in the future, and (6) were available to participate in a 90-min focus group conducted in English via Zoom.

Purposive sampling was used to ensure diverse representation in terms of race and geographic region, with the intention of capturing varied perspectives on race and racism. This included intentionally reaching out to an anti-racism group to incorporate insights from White individuals actively engaged in anti-racist work. Recruitment strategies included distributing flyers via social media (X, formerly known as Twitter and Facebook) and by contacting community organizations (e.g., YMCA, civic groups, and universities), including one organization focused specifically on anti-racism for White people. The study team sent emails to the organizations to request they share the information with their listservs and on their social media platforms. Those interested were invited to complete an online screening survey to collect the eligibility requirements, as well as regions of residence (captured by state on the form), contact information, and their availability to participate at the scheduled locations and times. This process provided a consistent and transparent format for the screening process. When potential participants met eligibility criteria and availability for the time of the focus group, they were contacted to confirm their participation.

This study was approved by the University of California, San Francisco Institutional Review Board (18–24,593). Women who identified as Black or White were recruited between June and August 2021. A total of six focus groups were conducted with 30 participants: three with Black women (*n* = 15 total; group sizes of 5, 6, and 4) and three with White women (*n* = 15 total; group sizes of 5, 3, and 7). All focus groups were racially homogeneous, including race-matched trained moderators.

Specifically, among the Black participants, two focus groups were composed of women from the South (*n* = 5 and 6), and one included women from the Western US (*n* = 4). Two of the White focus groups (*n* = 5 and *n* = 3) consisted of participants affiliated with a national anti-racism organization, with members located in the Northeast and Western US. This organization engages White individuals in racial and economic justice efforts through over 150 local chapters across the US and Canada. The third White focus group (*n* = 7) included women from the Southern US who did not identify as being part of an organized anti-racism group.

A semi-structured focus group guide was developed based on a review of the literature, and the study’s research aims to explore participants’ perceptions of their experiences related to race, racism, intersectionality, and protective strategies. The subset of questions on the guide was as follows: *Race*—“How do you think your race plays a role in your daily life? Can you give us some examples of whether you think it does or does not?”; *Racism*—“If you experience racism, where is it coming from? Please explain.”; *Intersectionality*—“Thinking about racism, do you think there are other aspects of your identity that also impact your experiences? Please share.”; and *Protective strategies*—“What are some steps you have taken to protect yourself or someone else from the impact of racism?” and “What resources do you think could help protect someone from the impact of racism?”.

The 90-min focus groups were conducted via Zoom, with participants recruited throughout the US Demographic information was collected using an online survey after the focus group. All participants received a $50 gift card. Focus group moderators used the same developed guide, which allowed for comparison of similarities and differences in experiences and perspectives of women across the focus groups.

To prepare for analysis, the sessions were audio-recorded, transcribed, and de-identified. The research team developed a codebook based on the categories outlined in the semi-structured focus group guide, which included questions related to race, racism, intersectionality, and protective strategies. Additional codes were added inductively as new concepts emerged from the data. NVivo was utilized for data management. Therefore, one team member coded the data in NVivo, while at least two other team members were present for coding of each of the six transcripts. This collaborative approach was chosen to facilitate discussions and ensure complete consensus on the most accurate categorization of the data. This collaborative coding process enhanced analytical rigor and allowed for immediate reflection on group dynamics and emergent insights.

Once all transcripts were coded, the team generated coding reports in NVivo. These reports are outputs that compile all text segments associated with each individual code. To enable comparative analysis, coding reports were disaggregated by focus group type: Black women, White women, and White women who were members of an anti-racism group. These reports allowed the team to examine how different themes were expressed across participant groups through an intersectional lens that took into account both gender and race. Using a thematic analysis approach guided by Braun and Clarke’s framework [29], the research team reviewed the coding reports through a series of iterative meetings. Key themes were identified and refined by examining patterns both within and across participant groups, with attention to areas of convergence and divergence in participants’ experiences. This approach facilitated an exploration of how racism and resistance were shaped by intersecting identities, particularly race and gender. Final themes were confirmed through team discussions to ensure consensus on the thematic structure [29].

In terms of positionality, the data collection and analysis team consisted of women who identified as Black, White, and Asian and recognized how their own lived experiences and perspectives shaded the analysis process. The authors have expertise in research examining race and ethnicity, which informed the initial analytical framing and grounding in current literature; however, they intentionally centered participants’ voices to avoid imposing assumptions and to ensure the analysis remained rooted in the lived experiences shared during the study. Throughout the multiple discussions about the data, the study team members were guided by their cultural knowledge and consistently acknowledged existing biases or assumptions throughout the process.

## Results

### Participants

The study included 15 Black participants (50%) and 15 White participants (50%). The mean age of the participants was 35.7 years old. Most participants were highly educated, with 80% having a bachelor’s or graduate degree. They represented various parts of the USA with 60% from the South, 23% from the West, and 17% from the Northeast (see Table 1 for breakdown by group).

**Table 1:** Study participants’ demographics

Demographic category	Total participants (*n* = 30)	Black women (*n* = 15)	White women (*n* = 7)	White women in anti-racism organization (*n* = 8)
**Age (in years)**	Mean: 35.7	Mean: 37.1	Mean: 24.4	Mean: 41.8
20–24	3 (10%)	0 (0%)	3 (43%)	0 (0%)
25–29	4 (13%)	1 (7%)	4 (57%)	0 (0%)
30–34	5 (17%)	2 (13%)	0 (0%)	2 (25%)
35–39	9 (30%)	7 (47%)	0 (0%)	2 (25%)
40–44	6 (20%)	5 (33%)	0 (0%)	3 (38%)
45–49	0 (0%)	0 (0%)	0 (0%)	0 (0%)
50–54	3 (10%)	1 (7%)	0 (0%)	1 (12%)
55 +	0 (0%)	0 (0%)	0 (0%)	1 (12%)
**Race *N* (%)**				
Black	15 (50%)	15 (100%)	0 (0%)	0 (0%)
White	15 (50%)	0 (0%)	7 (100%)	8 (100%)
**Education *N* (%)**				
Some College	6 (20%)	3 (20%)	2 (29%)	0 (0%)
Bachelor’s Degree	9 (30%)	3 (20%)	3 (43%)	3 (38%)
Graduate Degree	15 (50%)	9 (60%)	2 (29%)	5 (62%)
**Region of residence** ***N* (%)**				
West	7 (23%)	4 (27%)	0 (0%)	3 (38%)
Northeast	5 (17%)	0 (0%)	0 (0%)	5 (62%)
South	18 (60%)	11 (73%)	7 (100%)	0 (0%)

### Themes

#### Dynamic Between Responsibility and Privilege

### Theme Explanation

This theme captures how participants navigated responsibility and privilege in relation to their racial identity, specifically Black participants described a burden to protect themselves and others, while White participants reflected on using their privilege to act against racism.

Black participants reported a responsibility to protect themselves and other Black people in their community. They expressed that “feeling unsafe” necessitated a responsibility to act. The diminished feeling of safety was based on events such as the murder of George Floyd, as well as living with a constant realization that they and their family members were perceived as a threat, especially their Black husbands and sons. One participant said, “I remember like four years ago I was pregnant with my son, and one of the first things that I thought when I found out his sex was that, ‘Oh my gosh, I have to raise a Black male in America’” (Black FG3). Another participant reflected about her child, “How do I make sure my child is safe in this world related to their experiences as children and being villainized and seen as a threat” (Black FG1).

Black participants reported the uncertainties and stress of navigating spaces where they are the racial minority and the need for the non-minoritized groups to take responsibility for authentic relationships. Referring to the pressure, one participant said, “It is a burden and a stressor because again, at the base, we’re all human. We’re all having human experiences. But I don’t always feel like I can be less than strong or less than a star performer, whereas I feel like other people or other races or genders are able to have a bad week or mess up” (Black FG2).

Being the only Black person in professional settings contributed to this stress. One participant shared, “[I work] at a predominately White institution and I’m the only Black person in my department or in a lot of the spaces that I’m in, it’s very prevalent for me that I’m Black and I’m a Black woman” (Black FG1). Another added:

[Some coworkers are] just trying to figure you out and tip-toeing around you and just not being themselves. I don’t know. It’s difficult interacting with those kinds of co-workers because you’re like ‘Hey, just be yourself. Get to know me for me. You don’t have to be afraid.’ So I feel like that’s a daily struggle sometimes at work interacting with others. And then just realizing that some, this may be their first interaction honestly with a person of color (Black FG1).

Some White participants believed that they had a responsibility as well—to educate themselves and others about discrimination and act on behalf of marginalized groups. White participants stated that they were uneducated before or felt like they were asking stupid questions, but they felt compelled to make it better for the next generation. One participant expressed that she felt she had a responsibility to educate other White people and advocate for people of color. She recognized her position was one of privilege and could be more forceful and face less backlash compared to minoritized counterparts: “[I use] my Whiteness as a steamroller to push for changing things. So that people who are leading the direction of it weren’t the ones who were having to be more forceful or intense and deal with backlash” (White FG5 – Anti-Racism Group). Another participant expressed how her self-realization catalyzed her to action: “[I needed to understand] my Whiteness. …every Sunday I’ve been going to a protest. It’s like church – showing up in a physical way” (White FG4 – Anti-Racism Group). Some participants from Black and White focus groups reported feeling exhaustion and depression from the frustration of race relations in the country.

Many White participants discussed the role of privilege in their lives. One participant said, “the color of my skin has allowed me to live an easier life” (White FG6). Another participant expressed that she internalized racial superiority: “[I was] socialized to believe that I’m more competent or more entitled to certain things and more entitled to my safety” (White FG4 – Anti-Racism Group). Some participants indicated that privilege gave White people the ability to selectively engage and disengage in the fight for racial equality including the emotional turmoil it can cause: “Wow, as White people we really get to opt in and out of our rage, where people of color who are directly affected by these events, they don’t have that option” (White FG4 – Anti-Racism Group).

Some White participants shared explanations for being excluded to make space for people of color. One participant said:

As a White person, you might be asked to not participate in certain events or be specifically excluded. But I think of that usually as a way to create a safe space for people of color. And so take also like affirmative action. Not even giving, but equalizing the playing field which means that White people who would have formerly had access to a space, not as many might have access to that space anymore (White FG5 – Anti-Racism Group).

Another participant shared an experience of feeling excluded based on being White and stated, “One of my classes was how to teach African American students or how to teach students of every other race, but there was never a class on how to teach a White child. …Why are we not being taught how to teach a White child too?” (White FG6). One participant stopped at a gas station, and someone said, “you shouldn’t be here. And I was like, Why? And he was like, because you’re White” (White FG6), and the participant shared that the sentiment moved beyond exclusion to fear.

## Differing Experiences of Womanhood

### Theme Explanation

This theme explores how participants navigated the intertwined influences of race and gender in shaping their identities and daily experiences. Black women reported compounded pressures tied to racialized stereotypes, while White women described gender-based challenges often buffered by racial privilege. This theme reflects how womanhood is not experienced uniformly but is deeply shaped by racial positioning.

Our Black participants reported that they identify as “Black women” vs. only Black or only woman. That dual identity was salient and is impacted by how society views them, specifically within the lens of the “strong, Black woman.” One participant shared, “The one thing that sticks out to me is the stereotype that Black women are strong, we can handle it all, and we can get it done. …I don’t feel that I have that liberty to have a bad day or turn in my things late or just not show up to a meeting because I’m mentally burned out or whatever” (Black FG2).

Another stressor was the hyper-sexualization of Black women and girls. One participant stated:

It’s kind of the hyper-sexualization of Black women. And I think about it a lot because I have a young daughter. She’s a teenager now and her body is growing and developing, and she’s shapely. And I find myself encouraging her to cover up because I don’t want people gawking at her and I don’t want people assuming that she’s older than she is, because in my mind, she’s still my baby. But to others, she’s a young lady, a young woman. But I don’t want her over-sexualized. She’s 13, That’s an intersection that I feel myself being super protective over my child. And even with myself, I’m super aware of what I wear (Black FG2).

Misogyny and patriarchy were salient pressures reported among the White participants. One participant said her gender presents problems but not her Whiteness: “My gender is where I feel more of a problem. My Whiteness just allows me to come and go and opt out of things” (White FG4 – Anti-Racism Group). Some women also shared that their gender contributes to them not feeling safe, like at night in parking lots.

A few White participants reported that they have special societal advantages as women. One woman reported about the effect of her tears: “I feel with people in authority whether it’s at college or the police or anything like that I can get away with a lot…if I get pulled over and I just cry, they’ll probably let me go or I can just not get in trouble for a lot of things, because of both my ethnicity and my gender” (White FG6). Another participant shared that they have it easier, “especially just because we’re White girls” (White FG6).

## Self-Protection from Racism and Allyship from Racism

### Theme Explanation

This theme highlights the strategies participants used to protect themselves or support others—Black participants emphasized community care and self-preservation, while White participants described efforts toward allyship and advocacy.

Black participants reported the importance of protecting themselves and their community from racism. They reported many issues that should be addressed: eliminating barriers for resources, making housing equitable, and ensuring that there is Black representation in powerful positions. The participants also reported that people should know their rights, serve as an observer/witness of situations, and be “a pillar in the community” (Black FG3). Black participants also insisted upon the need for self-care—from social media breaks to prayer and therapy.

Both Black and White participants indicated the need for authentic conversations within their own racial group and across groups. One White participant said, “I think that it has been so helpful to me personally to have a group of White people to work all our sh*t out with together. And has definitely made my capacity for dealing with overt racist White people so much stronger and my ability to be able to engage with those people which I believe is my responsibility” (White FG5 – Anti-Racism Group). White participants reported the importance of participating in organizations that focus on dismantling racism, being present at events that support people of color, and supporting marginalized groups, such as Black Disability Coalition, Asian Solidarity Collective, and individual GoFundMe pages.

## Discussion

This paper provides a nuanced perspective of how Black women, White women, and White women from an anti-racism group perceive race and racism in their lives and considerations of how these dynamics intersect for women across racial groups. The study participants discussed the dynamic between responsibility and privilege. Black women expressed protecting themselves and family, White women from an anti-racism group shared the importance of educating themselves and others, White women from all groups discussed the role of privilege, and two White participants reported feeling excluded based on their race. Another theme was differing experiences of womanhood by racial identity. Specifically, Black participants reported hyper-sexualization of Black girls and women. Some White participants stated that gender was a more salient identity than their race and were impacted by pressures related to misogyny and patriarchy. Other White participants also expressed that there were some advantages of being White and a woman, such as crying to avoid reprimand. The theme of self-protection from racism and allyship from racism elucidated the importance of Black women protecting themselves and community from racism, and allyship required both Black and White women to have authentic conversations. White women from the anti-racism group also reported the need to support marginalized groups with presence and funding.

### Dynamic Between Responsibility and Privilege

Black participants expressed a deep sense of responsibility to protect themselves and their communities in response to persistent racial threats. This aligns with prior research on the Superwoman Schema (SWS), which describes how Black women cope with gendered racism by appearing strong, emotionally suppressed, and self-reliant [30, 31]. While this coping mechanism can be adaptive, it is also associated with poor mental and physical health outcomes [31, 32].

This study adds to existing research by exploring how White participants navigate privilege and responsibility. Two focus groups included members of anti-racism organizations, while another consisted of White participants from the South without such affiliations. Those engaged in anti-racism work more frequently expressed awareness of systemic racism and a sense of accountability, which aligns with prior findings [33, 34].

Across all White groups, many participants acknowledged White privilege led to “an easier life” and discomfort with racial injustice, suggesting that broader societal discourse may influence awareness, while anti-racism involvement may deepen it. This recognition diverges from prior studies that emphasize colorblindness and denial as common strategies White individuals use to deflect responsibility [35–37].

### Differing Experiences of Womanhood

While all women face societal challenges due to gender, including both hostile and benevolent sexism [38], race significantly shapes how these challenges are experienced. Black women described how the intersection of race and gender heightened the pressures they faced, particularly through stereotypes such as the “strong Black woman” and the hyper-sexualization of Black girls. These findings align with prior research showing that Black women are often expected to embody resilience, suppress emotion, and shoulder responsibility, frequently at the expense of their own well-being [19–22].

Although gender-based mistreatment is a common experience among women, race often shapes how such mistreatment is perceived, navigated, and responded to. A qualitative study of 31 participants found that while both Black and White women reported experiencing gender-based discrimination, White women were more likely to view their race as a resource in managing these challenges [16]. This pattern was echoed in our findings when one White participant shared that she avoided a speeding ticket by crying, reflecting research on the use of tears by some White women to assert victimhood while minimizing the personhood of others [39]. These accounts underscore how racial identity influences the perception and impact of gendered experiences, even among women who share the same gender identity.

### Self-Protection from Racism and Allyship from Racism

Participants described various strategies for navigating racism, ranging from self-protection to allyship. Some White participants, particularly those in anti-racism groups, expressed a desire to improve conditions for future generations. This aligns with a USA Today/Ipsos poll showing over half of US parents support teaching children about ongoing racism—though only 37% of White parents supported Critical Race Theory in schools, compared to 83% of Black parents [40].

These generational dynamics reflect broader patterns of racial socialization. After George Floyd’s death, Black parents reported increased worry and more frequent conversations with their children about racial bias [41]. In contrast, White parents were less likely to discuss Whiteness, more likely to use colorblind language, and generally less concerned about their children experiencing or contributing to racism [41]. Similarly, few White mothers in Minneapolis discussed systemic racism after Floyd’s death [12]. This echoes participants’ comments about the privilege of being able to “opt in and out of our rage.”

However, a small subset of White participants framed themselves as victims, expressing feelings of exclusion or reverse discrimination. For instance, one participant questioned why there are no classes on “how to teach White children.” Such statements reflect a lack of recognition that Whiteness is often the unspoken norm within US institutions [36, 37, 42] and reveal how feelings of discomfort or perceived loss of status can be misinterpreted as oppression [10].

In line with previous literature on the importance of racial and gender solidarity, many Black and White Women in these focus groups discussed how they see positive impacts in their lives from having substantial conversations with other women from their own communities, which is supported by past literature as friendships and community provide women with the support they need through emotional understanding and empowerment [16].

## Strengths and Limitations

The study is valuable in that it offers qualitative research from Black women, White women, and White women from an anti-racism organization. This study’s data served to provide context of the participants’ perspectives and not as population-level representation. In the future, it would be helpful to conduct more focus groups across the three categories to investigate with a larger sample the breadth of diverse experiences, including a deeper exploration of the topic of allyship. Even though some White participants discussed allyship, ineffective allyship—characterized by failures to acknowledge privilege or address biases—can create psychological distress for marginalized groups, underscoring contradictions between intent and impact [43, 44].

The use of Zoom for focus groups enabled participation from individuals across the USA, with participants engaging via video and audio. While virtual sessions increased accessibility, they may have limited rapport-building and reduced the ability to observe non-verbal cues, potentially affecting participant disclosure. Additionally, the virtual format restricted participation to individuals with internet access. Most focus group participants were also highly educated. While higher socioeconomic status can buffer against certain material and psychosocial stressors [45], some research indicates that highly educated Black women are more likely to report racial discrimination than those with lower educational attainment [46, 47]. In the future, in-person focus groups could help engage participants from a wider range of educational backgrounds.

Recruitment relied on social media with mixed results and community-based organizations, which were responsive to outreach efforts and helped disseminate information. However, this approach may have introduced bias, as individuals who were more civically engaged or already interested in racial equity may have been more likely to participate. To capture a broader range of perspectives, future studies would benefit from larger sample sizes, more focus groups, and greater socioeconomic diversity. This study provides a valuable foundation for expanding qualitative and quantitative research on these issues.

## Conclusion

The findings of this study suggest that many Black participants feel that their only recourse for protecting themselves and their communities from the effects of racism is to rely solely on their own efforts. A study on faculty of color also described the term cultural taxation, or the expectation that faculty of color would be the departmental voice of diversity, while their White colleagues were not [39]. It reflected an assumption that diversity-related issues were not important for White faculty because their lives were not affected by “those kinds of issues” [48]. While some White participants were very vocal in describing how it was their responsibility to educate other White people about discrimination and act as advocates, all White participants did not share this view. Others felt that it was they who were victims of discrimination based on their race. Studies have identified the deep importance of family socialization, and how White parents can act as carriers of racism to their children unless they engage them in conversations grounded in anti-racism and equity [10]. Further research could explore more deeply what White participants did in their efforts to educate themselves and other White people about racism, as well as how they acted as advocates for people of color and how everyone can help to combat racism. This study hopes to spur further investigations into the role of racism in daily life for White and Black Americans. Understanding experiences, perspectives, and proposed solutions can help spark needed change to create a culture of empathy, reflection, and shared responsibility.

## Data Availability

IRB only provided permission to the study team to have access to the transcripts.

## Code Availability

No quantitative coding in this project.
